# Preliminary analysis of effects of the 2006 Turin Winter Olympic Games on air quality

**DOI:** 10.1371/journal.pone.0205975

**Published:** 2018-10-18

**Authors:** Antonella Senese, Michele Valenti, Vincenzo Senese

**Affiliations:** 1 Department of Environmental Science and Policy, Università degli Studi di Milano, Milan, Italy; 2 Studio SBConsulenza, Polo Tecnologico, Pavia, Italy; National Sun Yat-sen University, TAIWAN

## Abstract

This paper presents preliminary results about Turin’s air quality before, during and after the realization of the infrastructure projects for the Turin 2006 XX Winter Olympic Games. We compared the 3-year *in-operam* (work in progress) period (i.e. 2003–2005, when all infrastructures needed for the organization were built) with the periods before (*ante-operam*) and after (*post-operam*): 2000–2002 and 2006–2008, respectively. In particular, we analyzed the concentrations of the primary pollutants (nitrogen dioxide, carbon monoxide and fine particulate matter, PM_10_) in Turin and Milan. In this way, we could use the measurements from Milan as a control dataset for comparison with the atmospheric pollution conditions in Turin. We found that infrastructural work for the Olympic Games in Turin affected NO_2_ and PM_10_ atmospheric concentrations, determining a peak in the average values during the *in-operam* period (probably due to caterpillar tractor emissions and excavation). This pattern did not emerge from the Milan data, where a decreasing trend can be seen between the *ante-operam* and the *post-operam* periods. On the other hand, a negative effect on CO levels was not observed: the decreasing trend, more evident in Turin compared to Milan, can be linked to the expansion during the same period of limited traffic areas created to facilitate the infrastructural work.

## Introduction

Turin (Italy) hosted the 2006 Winter Games of the XX Olympic Games from February 10^th^ to 26^th^. One of the main issues related to the organization of Olympic Games is air pollution. More in general, in urban environments, particular social events can play a negative role by increasing air pollutant levels: festivals and concerts, international meetings and sporting events ([1], [2]). The Olympic Games are among the events with the greatest impact ([3], [4], [5]). In fact, whether played or watched, athletic endeavors have the potential to produce huge environmental “footprints” in terms of their use and abuse of natural resources [6]. Ski slopes, for instance, disrupt fragile alpine ecosystems, while snowmobiles spew exhaust fumes into the air. Golf courses sprawl across the land, and consume large amounts of pesticides and water, while parking lots for stadiums and arenas produce vast paved surfaces. In addition, major sports events use energy, emit greenhouse gases, and produce voluminous trash. For example, the 2006 Super Bowl in Detroit produced 500 tons of the greenhouse gas carbon dioxide (from transportation and natural gas and electricity consumption), while the 2004 Summer Olympics in Athens produced half a million tons in two weeks (roughly comparable to what a city of 1 million people would emit over a similar period [6]). Each match during the 2006 World Cup used up to 3 million kilowatt-hours of energy (similar to the annual consumption of 700 European households) and produced an estimated 5–10 tons of trash.

In the available literature, studies usually examine the short-term impacts—that is, in the course of the Games—without considering any effects of the previous organizational phases. For instance, [7] investigated the effects of the measures implemented by the Beijing municipal government (China) to reduce urban motor vehicle emissions only during the 2008 Olympic Games. [5] examined the role of sources outside Beijing in summertime Beijing air pollution levels (that is, earlier than the 2008 Olympic Games) in order to contribute to the regional air quality management studies and new emission control strategies to ensure that the air quality goals for 2008 were met. [8] analyzed the concentrations of benzene and formaldehyde in air in two venues of the 2006 Turin Olympic Games (Turin and Pragelato), aiming to conduct an environmental health survey related to this important event. In this case, the study period was one year before the sport event.

The most attention has been paid to the impacts on human health. In fact, the goal of air quality policies is to protect public health by implementing regulatory measures or providing economic incentives that help to reduce the public’s exposure to atmospheric pollutants. For example, [9] described traffic changes in Atlanta (USA) during the 1996 Summer Olympic Games and concomitant changes in air quality and childhood asthma events. [10] considered the effects on asthma in adults during the 2008 Summer Olympic Games in Beijing. [11] analyzed data from the previous five Olympic Games obtained from athletes seeking to use inhaled β2 adrenoceptor agonists (IBA), in order to identify those athletes with documented asthma and airway hyper-responsiveness (AHR) and the evolution of their illness. In fact, breathing polluted or cold air is considered a significant etiological factor in some but not all sports. [12] described the available evidence of the effect of air quality on performance, especially as it pertains to athletes with asthma.

**Fig 1:**
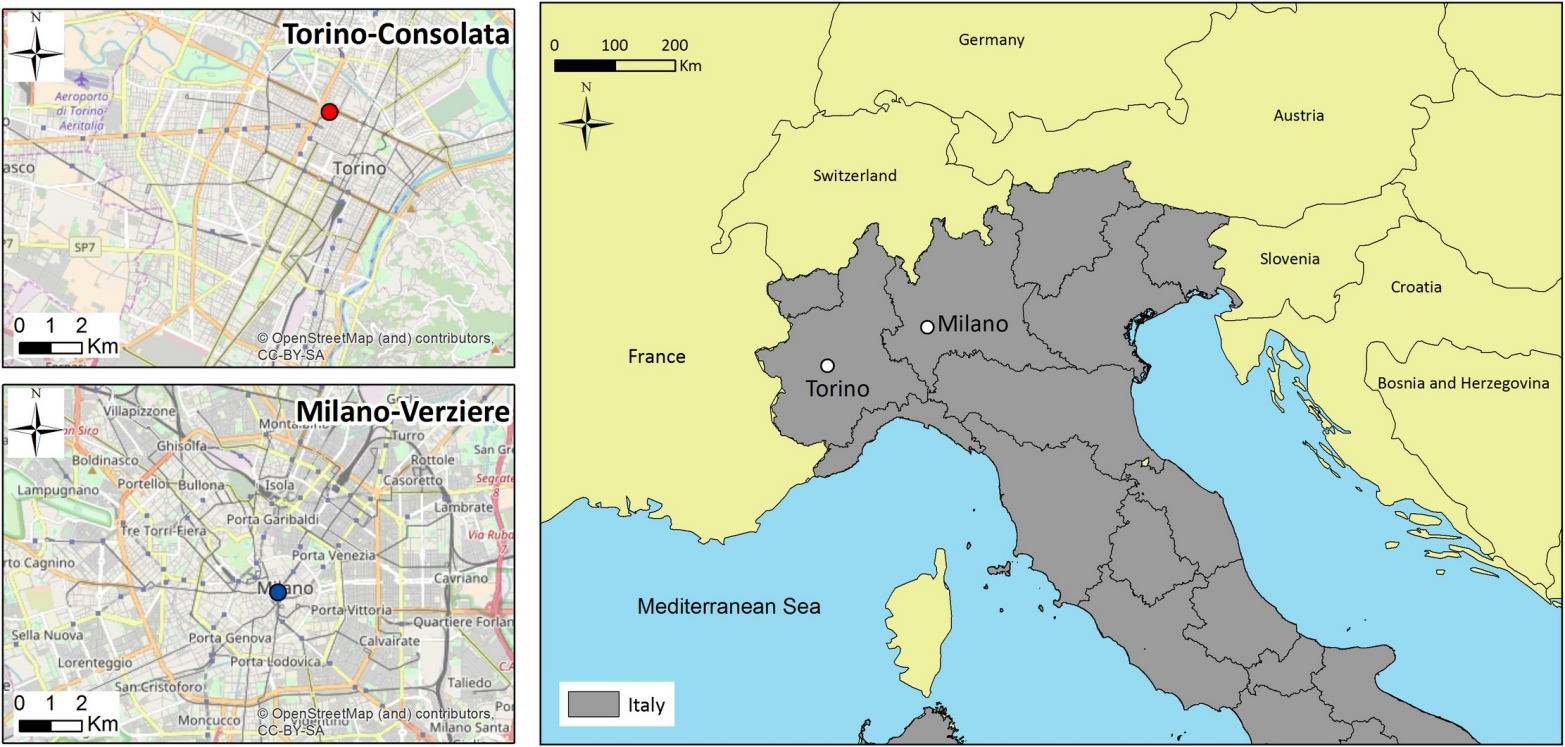
Topography of Northern Italy (the Po Valley) and the geographical location of the city air quality monitoring stations (Turin and Milan).

The present study attempts to examine this important topic in the context not only of the 2-week period of the Turin Winter Olympics but also the 3-year period when all infrastructures needed for the organization were built (2003–2005). For this reason, we compared the *in-operam* period (i.e. 2003–2005) with the ones before (*ante-operam*) and after (*post-operam*): 2000–2002 and 2006–2008, respectively. In particular, we analyzed the atmospheric concentrations of the primary pollutants at ground level [13]: nitrogen dioxide (NO_2_), carbon monoxide (CO) and PM_10_ (breathable particulate matter with an aerodynamic diameter < 10 μm). These molecules and compounds are included in the list of the parameters to be considered for analyzing air quality defined by the European Community in Directive 2008/50/CE. In particular, we chose these three parameters alone, as they are the only ones with uninterrupted data sets for the period examined (9 years). The study was conducted analyzing the information for Turin and Milan. We used the measurements from Milan as a control dataset for comparison with the atmospheric pollution conditions in Turin (venue of the Olympic Games). The comparison is sustainable since these two cities are both large metropolitan areas in northern Italy (Fig 1) and they are similar in terms of air pollutant sources and levels. Moreover, in order to perform a more exhaustive analysis, we compared the pattern of nitrogen dioxide, carbon monoxide and fine particulate matter during the two weeks of the Turin Winter Olympics (from 10 to 26 February 2006) against normal days unaffected by the Games.

The leading sources of nitrogen dioxide (NO_2_) are traffic and, to a lesser extent, industry, shipping and households. High nitrogen dioxide levels, combined with ultrafine particles and other oxidants, have become one of the major air pollution problems in urban areas all over the world [13]. Nitrogen oxides are one of the main components of the pollution mix classically referred to as “photochemical smog”. Short-term nitrogen dioxide concentrations may vary considerably within cities and from time to time during the day and night. In addition, average concentrations depend on the distance of the measurement station from main roads [13].

Carbon monoxide (CO) is one of the most common and widely distributed air pollutants. It is a colorless, odorless, and tasteless gas that is poorly soluble in water [14]. The annual global emissions of carbon monoxide into the atmosphere have been estimated to be as high as 2600 million tons, of which about 60% are from human activities and 40% from natural processes [15]. Anthropogenic emissions of carbon monoxide originate mainly from incomplete combustion of carbonaceous materials. The largest proportion of these emissions are produced as internal combustion engine exhaust, especially from motor vehicles with gasoline engines. Other common sources include various industrial processes, power plants using coal, and waste incinerators. Petroleum-derived emissions have greatly increased during the past few decades [16]. Some widespread natural non-biological and biological sources, such as plants, oceans and oxidation of hydrocarbons, give rise to the background concentrations outside urban areas [14].

Particulate matter (PM) in urban and non-urban environments is a complex mixture with components having diverse chemical and physical characteristics. Newer research findings continue to highlight its complexity and dynamic nature: different characteristics (size, other physical features, chemical composition and source) of PM may be relevant to different health effects, and the formation of PM may be either primary or secondary, and then continues to undergo chemical and physical transformation in the atmosphere [13]. For current regulatory purposes, PM has been classified by aerodynamic diameter, as size is critical in determining the likelihood and site of deposition within the respiratory tract. In 1987, the US EPA established a standard for PM at less than 10 μm in aerodynamic diameter (PM_10_) [17]. PM_10_ includes those inhalable particles that are sufficiently small to penetrate to the thoracic region. In urban areas, these particles typically contain resuspended dust from roads and industrial activities, and biological material such as pollen grains and bacterial fragments. In addition, they include the earth’s crustal materials such as wind-blown dust from agricultural processes, uncovered soil, unpaved roads or mining operations. Traffic produces road dust and air turbulence that can re-entrain road dust near roadways. Near coasts, evaporation of sea spray can also produce large particles. Coarse particles may also be formed from the release of non-combustible materials in combustion processes, i.e. fly ash.

Global background concentrations of carbon monoxide range between 0.06 and 0.14 mg/m^3^ (0.05–0.12 ppm). During the early 1980s, there was an annual 1–2% increase in non-urban tropospheric concentrations of carbon monoxide, whereas between 1989 and 1992 concentrations began declining rapidly [14].

An overview of typical annual average concentrations of NO_2_ and PM_10_ in selected cities around the world was presented by [13]. Rural background concentrations of nitrogen dioxide in industrialized countries have been measured at around 15–30 μg/m^3^. In urban areas, NO_2_ concentrations exceed 40 μg/m^3^ as an annual value (WHO’s 2000 air quality guideline, [14]) in many of the larger cities on all continents. The annual average NO_2_ concentrations in major European cities were reported in 2002 to range from 14 μg/m^3^ in Iceland to 44 μg/m^3^ in France [18]. Regarding PM_10_, in urban areas, the average concentrations were 26.3 μm/m^3^ (annual) and 43.2 μm/m^3^ (daily), while in rural areas the concentrations were 21.7 μm/m^3^ and 38.1 μm/m^3^ (annual and daily, respectively). In Europe, the typical range was 15–60 μm/m^3^. Focusing on Turin, the mean value is about 60 μm/m^3^.

### Study area

Field studies were conducted at two sites in and outside the Olympic Games urban area: Turin and Milan, respectively, both located in the Northern part of Italy. The two sites are shown in Fig 1 and are described in the following paragraphs.

The territory of the Province of Turin (known since 2015 as the Metropolitan City of Turin), 6330 km^2^, 315 municipalities, 13 Mountain Communities, stretches from the Cozie Alps, descending towards the plain in a range of valleys: the valleys of the Pinerolo area, the Val di Susa, and the Lanzo and Canavese valleys. The city of Turin, with approximately 900,000 inhabitants, is the capital of Piedmont [19]. It is a city with a history of enormous importance: it was the capital of the Savoy Duchy, then of the Kingdom of Sardinia and, for a short time, of unified Italy. Piedmont's capital is today one of the most developed and technological industrial centers in Europe.

Unlike the previous Winter Olympics, in which the events took place in districts with limited distances between one another, the territory affected by the 2006 Turin Games was vast [20]. The municipalities hosting the Olympic infrastructures were separated by many kilometers: Turin-Sestriere = 102 km, Turin-Bardonecchia = 92 km, Turin-Oulx = 79 km, Turin-Pragelato = 101 km, Turin-Cesana = 91 km. In this broad panorama, more than 65 facilities were built for the 2006 Turin Olympics (pistes, villages for housing athletes and media workers, indoor sports arenas, ski lifts, …), and in order to connect them optimally, 25 km of new roads, 150 km of upgraded roads and two enhanced rail lines were built to make the distances compatible with Olympic standards and with the requirements of the Comité International Olympique (CIO) [21].

Therefore, the area involved was very large: about 773,888 m^2^ of construction sites plus 175 km of roadwork (new and upgraded) and 565,300 m^3^ of new snow storage basins for the ski slopes. It is highly likely that this led to an alteration of the air quality [21]. For this reason, the period when these projects were carried out (i.e. *in-operam*) was the subject of this study, compared with the control periods (*ante*- and *post-operam*).

In addition to the beginning of construction works, our *in-operam* period coincides with the establishment of several limited traffic areas (the so-called ZTL, according to law 523 of 2004), that were enlarged during the following years.

Milan is the capital of Lombardy and the second most populous city in Italy after Rome, with the city proper having a population of about 1,400,000 [22]. Its continuously built-up urban area (that stretches beyond the boundaries of the Metropolitan City of Milan) has a population estimated to be about 5,270,000 over 1,891 km^2^, ranking 4^th^ in the European Union [23]. It hosted the Universal Exposition in 2015, but the relative infrastructure building activities started after 2009, so this did not affect the atmospheric pollution data we used to compare against the Turin dataset. The work on the two new subway lines (M4-blue and M5-lilac) can also be disregarded as it began in 2008. The policies to reduce air pollution by avoiding road traffic in the town center were established in 2008 and in 2012 (the so-called Ecopass Area and Area C, respectively). Again in this case, these measures were adopted after the end of our study period and they did not affect the datasets used.

### Data and methods

We analyzed chemical datasets acquired by the stations of Turin-Consolata and Milan-Verziere, both Urban-Traffic stations (i.e. located in high density urban areas where the main source of pollution is road traffic). Data from Turin-Consolata (45° 4’ 47” N, 7° 40’ 45” E, altitude: 243 m) can be downloaded from the environmental portal of the Regional Air Quality Monitoring System (Aria Web). The station of Milan-Verziere (45° 27’ 48” N, 9° 11’ 43” E, altitude: 116 m) belongs to the ARPA Lombardia (Lombardy Regional Agency for the Environment) network. At both stations, all data are recorded every hour, except for PM_10_ which is evaluated using daily samples.

Nitrogen dioxide is measured using chemiluminescence. The infrared absorption technique is used for sampling carbon monoxide. PM_10_ is measured by means of gravimetric and continuous gravimetric methods.

Hourly data (for CO and NO_2_) and daily data (for PM_10_) obtained from Regional Environmental Protection Agencies registries, were validated and sorted to create three separate elementary data vectors (one for each of the three-year periods selected: *ante-operam*, 2000-2001-2002; *in-operam*, 2003-2004-2005; *post-operam*, 2006-2007-2008). We obtained three hourly datasets (of 26,280 data each) for both NO_2_ and CO and three daily datasets (of 1095 data each) for PM_10_. Each data vector was analyzed to define the statistical distribution of values. Validated values were divided into 15 increasing classes. For each data vector were calculated the histograms of data distribution among the defined classes. We calculated a lognormal distribution for all the datasets because it is well known for describing environmental concentrations of pollutants [24]. It is an asymmetric distribution since it is skewed towards zero values with a longer right tail than the Gaussian distribution. In the environmental media (air, water, soil), low concentrations of pollutants appear more frequently, though there is a long series of higher values appearing less frequently.

Starting from experimental hourly and daily data, lognormal distributions were calculated for the three pollutants and for each of the three periods considered (*ante-operam*, *in-operam*, and *post-operam*). In a data vector of hourly or daily atmospheric concentrations, the frequency of the *x* concentration can be described by the following formula, if the data follow a lognormal distribution:

$$f\left(x\right)=\frac{e^{-\frac{{\left(\text{ln}\;x-\mu \right)}^{2}}{2\sigma^{2}}}}{x\sqrt{2\pi }\;\sigma }$$ (1)

where *f(x)* is the probability of the atmospheric concentration *x*, *σ* is the standard deviation of the transformed variable dataset *y* and *μ* is the average of the transformed variable dataset *y*, where *y = ln(x)*. The average value (*μ*_*x*_) and the standard deviation (*σ*_*x*_) of the variable *x* dataset (atmospheric concentrations dataset) are given by the formulas:

$$\mu_{x}=e^{\left(\frac{\sigma_{y}{}^{2}}{2}+\mu_{y}\right)}$$ (2)

$$\sigma_{x}=ln\left(\frac{\sigma_{y}{}^{2}}{\mu_{y}{}^{2}}+1\right)$$ (3)

where *μ*_*y*_ and *σ*_*y*_ are the average and the standard deviation, respectively, of the transformed variable dataset of *y* values.

The probability of the 15 concentration classes was re-calculated on the basis of the theoretical parameters of the lognormal distribution. The probability *P(i)* of the *i*^*th*^ concentration class is given by the formula:

$$P\left(i\right)=\int_{x=il}^{x=iu}f\left(x\right)dx$$ (4)

where *f(x)* is given by the Eq 1, *x* is the atmospheric concentration, *iu* is the upper limit of the *i*^*th*^ concentration class, and *il* is the lower limit of the *i*^*th*^ concentration class.

For each dataset, we calculated the lognormal parameters and we obtained statistical indicators to compare the evolution over time of the data series for each of the three selected pollutants. In this way, we obtained indicators describing air quality trends in the 9 years of the selected period (2000–2008) as regards Turin and Milan.

Finally, in order to verify the “goodness-of-fit” between observed and theoretical data and then to evaluate the reliability of the lognormal distribution, we applied the Chi-Square Test. The Chi-Square Test is a statistical hypothesis test that compares the sum of the squared differences between the empirical and the theoretical frequencies of each class of values (χ2) with the critical values of the Chi-Square table, r.

## Results

Concentration class ranges and frequencies of elementary data for the three selected periods for Turin (*ante-operam*: 2000-2001-2002; *in-operam*: 2003-2004-2005; and *post-operam*: 2006-2007-2008) are shown in Table 1 where statistical information of concentration data are present too. Almost for all data vectors of NO_2_ and CO, the valid data are higher than 95% (except for the *post-operam* NO_2_ value at 66%). Even if PM_10_ features slightly lower values (74–85%), these data can still be considered suitable for all the statistical analyses.

**Table 1:** Concentration class ranges and frequencies of elementary data for the three selected periods for Turin (*ante-operam*: 2000-2001-2002; *in-operam*: 2003-2004-2005; *post-operam*: 2006-2007-2008) and statistical information concerning the concentration data.

NO_2_ –Hourly Air Concentration—TURIN				CO–Hourly Air Concentration—TURIN				PM_10_ –Daily Air Concentration—TURIN			
Conc. Classes - (μg/m^3^)	*Ante-Operam* Frequency Nr. Data	*In-Operam* Frequency Nr. data	*Post-Operam* Frequency Nr. data	Conc. Classes - (mg/m^3^)	*Ante-Operam* Frequency Nr. Data	*In-Operam* Frequency Nr. Data	*Post-Operam* Frequency Nr. Data	Conc. Classes - (μg/m^3^)	*Ante-Operam* Frequency Nr. Data	*In-Operam* Frequency Nr. Data	*Post-Operam* Frequency Nr. Data
**0–22**	812	895	768	**0.0–0.6**	1155	1035	1844	**0–22**	15	4	17
**22–44**	4746	3596	3361	**0.6–1.2**	6437	10159	14210	**22–44**	100	67	152
**44–66**	8832	7130	4917	**1.2–1.8**	6080	6509	5794	**44–66**	193	169	220
**66–88**	6018	7397	4032	**1.8–2.4**	4393	3747	2470	**66–88**	142	191	180
**88–110**	2836	3981	2399	**2.4–3.0**	2844	1904	972	**88–110**	137	191	133
**110–132**	1205	1382	1008	**3.0–3.6**	1874	967	364	**110–132**	94	125	86
**132–154**	496	432	471	**3.6–4.2**	1083	501	119	**132–154**	54	80	64
**154–176**	190	121	220	**4.2–4.8**	754	234	0	**154–176**	27	57	30
**176–198**	74	42	78	**4.8–5.4**	420	120	0	**176–198**	24	31	16
**198–220**	24	19	34	**5.4–6.0**	261	50	0	**198–220**	12	13	10
**220–242**	15	7	24	**6.0–6.6**	160	33	0	**220–242**	4	1	3
**242–264**	10	1	6	**6.6–7.2**	120	11	0	**242–264**	0	0	1
**264–286**	1	0	1	**7.2–7.8**	65	12	0	**264–286**	1	0	1
**286–308**	2	0	0	**7.8–8.4**	52	4	0	**286–308**	2	0	3
**308–330**	0	0	1	**8.4–9.0**	22	0	0	**308–330**	0	0	0
**Nr. Data**	25262	25003	17320	**Nr. Data**	25773	25289	25883	**Nr. Data**	805	929	916
**Valid Data (%)**	96%	95%	66%	**Valid Data (%)**	98%	96%	98%	**Valid Data (%)**	74%	85%	84%
**Max**	346.0	253.0	321.0	**Max**	17.5	11.5	8.0	**Max**	296.0	225.0	306.0
**Mean**	66.6	70.4	69.3	**Mean**	2.0	1.5	1.2	**Mean**	88.5	96.8	83.4
**Std. Dev.**	29.9	28.6	33.6	**Std. Dev.**	1.4	0.9	0.7	**Std. Dev.**	45.2	41.4	44.3
**25**^**th**^ **percent.**	47.0	51.0	45.0	**25**^**th**^ **percent.**	1.1	0.9	0.7	**25**^**th**^ **percent.**	54.0	66.0	50.0
**Median**	62.0	69.0	65.0	**Median**	1.7	1.3	1.0	**Median**	78.0	93.0	75.0
**75**^**th**^ **percent.**	82.0	88.0	88.0	**75**^**th**^ **percent.**	2.6	1.9	1.4	**75**^**th**^ **percent.**	114.0	121.0	108.0
**95**^**th**^ **percent.**	122.0	120.0	131.0	**95**^**th**^ **percent.**	4.6	3.3	2.5	**95**^**th**^ **percent.**	178.0	175.0	165.5

**Fig 2:**
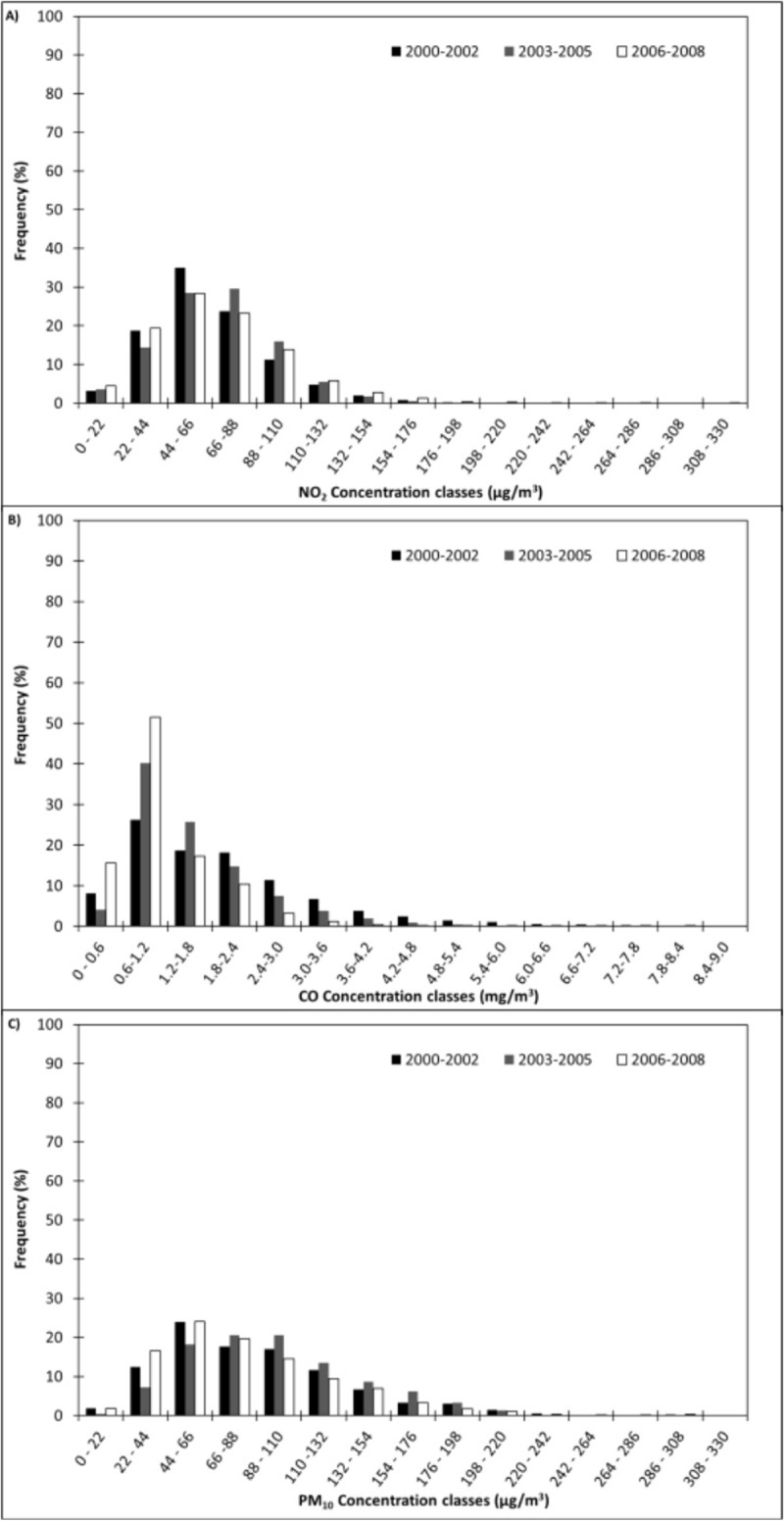
Frequency distribution obtained for each of the three data vectors (2000–2002; 2003–2005; 2006–2008) of each pollutant in Turin: NO_2_ (A), CO (B) and PM_10_ (C).

In Fig 2 are shown the histograms of frequencies obtained for each of the three data vectors (2000–2002, 2003–2005 and 2006–2008) of each pollutant. Validated values were divided into 15 increasing classes (see also Table 1). The peak is reached at 44–88 μg/m^3^, 0.6–1.2 mg/m^3^ and 44–110 μg/m^3^ for NO_2_, CO and PM_10_, respectively. As expected, an asymmetric distribution is found for all three pollutants, thus suggesting to correctly consider a lognormal distribution. The statistical parameters of calculated lognormal distributions are shown in Table 2.

**Table 2:** Parameters of calculated lognormal distributions related to Turin data: *f(x)* ≅ *log* *N* (*μ*_*y*_ *; σ*_*y*_*)*.

	*Ante-Operam* 2000-2001-2002			*In-Operam* 2003-2004-2005			*Post-Operam* 2006-2007-2008		
	Lognormal distribution	*μ*_*x*_	*σ*_*x*_	Lognormal Distribution	*μ*_*x*_	*σ*_*x*_	Lognormal Distribution	*μ*_*x*_	*σ*_*x*_
**NO**_**2**_	f(x) = logN (4.10; 0.47)	67.28	33.63	f(x) = logN (4.16; 0.48))	71.71	36.73	f(x) = logN (4.11; 0.54)	70.54	40.75
**CO**	f(x) = logN (0.50; 0.64)	2.02	1.45	f(x) = logN (0.27; 0.54)	1.51	0.88	f(x) = logN (0.04; 0.49)	1.17	0.60
**PM**_**10**_	f(x) = logN (4.35; 0.55)	90.11	53.93	f(x) = logN (4.47; 0.46)	97.52	47.40	f(x) = logN (4.28; 0.55)	84.27	49.99

**Fig 3:**
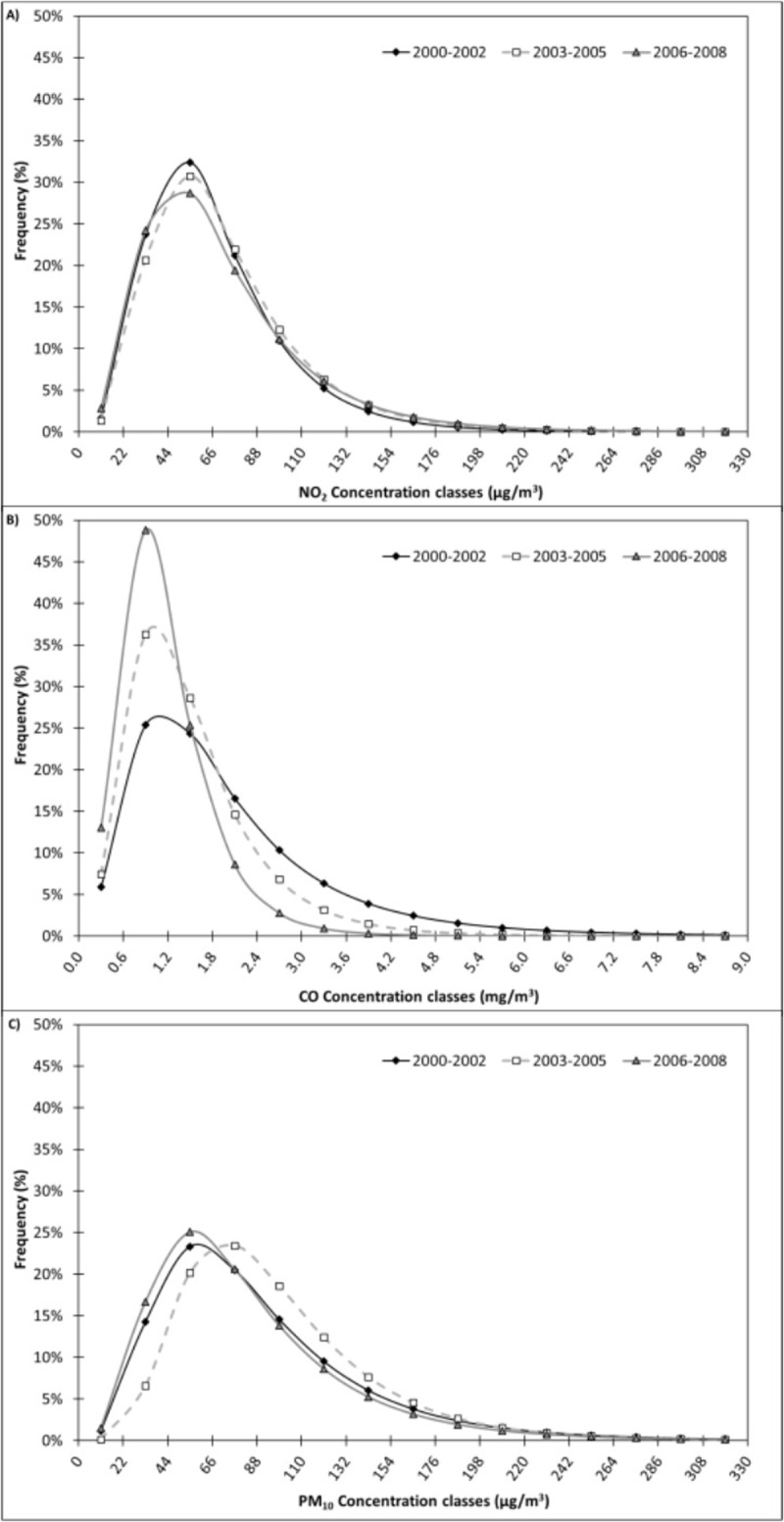
Re-calculated frequency distribution of NO_2_ (A), CO (B) and PM_10_ (C) sorted according to 15 concentration classes recorded in Turin in each of the three periods (*ante-operam*, *in-operam*, and *post-operam*).

By applying the Eq 4 to all the concentration classes it was possible to obtain the re-calculated frequency of each atmospheric concentration class and to plot the frequency of the 15 selected classes in each of the three periods (*ante-operam*, *in-operam*, and *post-operam*) obtaining the graphs of frequency distribution curves, as shown in Fig 3.

NO_2_ atmospheric concentrations show a higher average value (*μ*_*x*_) during the *in-operam* period (71.71 μ g/m^3^ vs 67.28 μg/m^3^ in the *ante-operam*, and 70.54 μg/m^3^ in the *post-operam*, Table 2). The lognormal frequency distribution curve of the *in-operam* dataset is slightly skewed towards higher values compared to the *ante-* and *post-operam* periods (Fig 3A). While the 0–40 μg/m^3^ class frequency is lower in the *in-operam* period (16.7% vs 19.4% in the *ante-operam* and 21.5% in the *post-operam*, Table 3) compared with the 70–160 μg/m^3^ class frequency (39.5% vs 35.6% in the *ante-operam* and 36.3% in the *post-operam*). An increasing trend of >160 μg/m^3^ class frequency can be observed from the *ante-operam* period (1.9%) to the *in-operam* (2.8%) and *post-operam* (3.7%). Even if the *post-operam* period shows decreasing average atmospheric concentrations, it is characterized by an increased frequency of lower values (<40 μg/m^3^) and isolated extreme concentration values (>200 μg/m^3^). This can be linked to the consideration that during the infrastructural works for the Olympic Games a more constant and higher emission level was present, together with reduced extreme and isolated concentrations (constant presence of caterpillar tractors and reduced road traffic emissive profile).

**Table 3:** Re-calculated frequency distribution of NO_2_, CO and PM_10_ sorted according to 5 concentration classes recorded in Turin in each of the three periods (*ante-operam*, *in-operam*, and *post-operam*).

NO_2_–Hourly Air Concentration– Lognormal Distribution–TURIN				CO–Hourly Air Concentration Lognormal Distribution–TURIN				PM_10_–Daily Air Concentration Lognormal Distribution–TURIN			
Conc. Classes (μg/m^3^)	*Ante Operam* Frequency	*In* *Operam* Frequency	*Post Operam* Frequency	Conc. Classes (mg/m^3^)	*Ante Operam* Frequency	*In* *Operam* Frequency	*Post Operam* Frequency	Conc. Classes (μg/m^3^)	*Ante Operam* Frequency	*In* *Operam* Frequency	*Post Operam* Frequency
**0–40**	19.4%	16.7%	21.5%	**0–1**	22.0%	31.0%	47.1%	**0–40**	11.7%	4.4%	13.9%
**40–70**	43.2%	40.9%	38.5%	**1–2**	40.0%	47.5%	44.1%	**40–70**	31.2%	26.8%	33.5%
**70–110**	27.4%	29.4%	26.3%	**2–5**	33.8%	20.9%	8.8%	**70–110**	30.9%	37.6%	30.2%
**110–160**	8.2%	10.1%	10.0%	**5–7**	3.0%	0.5%	0.1%	**110–160**	16.8%	21.6%	14.9%
**160–200**	1.4%	1.9%	2.3%	**7–10**	1.0%	0.1%	0.0%	**160–200**	5.1%	5.9%	4.2%
**>200**	0.5%	0.9%	1.4%	**>10**	0.3%	0.0%	0.0%	**>200**	4.3%	3.7%	3.2%

The maximum frequency of CO atmospheric concentrations is generally reached at the 0.6–1.2 mg/m^3^ class (Fig 3B). The highest frequency (48.8%) is recorded during the *post-operam* period, when CO frequency is more concentrated between 0.6 and 1.2 mg/m^3^. On the other hand, the *ante-operam* and *in-operam* periods show wider curves (between 0.0 and 3.6 mg/m^3^ and 0.0 and 3.0 mg/m^3^, respectively). Analyzing the CO trend from the *ante-operam*, to the *in*- and *post-operam* periods, there is a decreasing trend in the concentration values > 1.8 mg/m^3^ and an increasing trend in 0.0–1.2 mg/m^3^ classes. Average values of atmospheric concentrations clearly show a decreasing trend: 2.0 mg/m^3^ in the *ante-operam* period, 1.5 mg/m^3^ in the *in-operam* period and 1.2 mg/m^3^ in the *post-operam* period (Table 2). On the other hand, an increasing trend is present for the frequency of <1.0 mg/m^3^ classes (22.0%: *ante-operam*; 31.0%: *in-operam*; 47.1%: *post-operam*, Table 3). Higher concentration class (>7.0 mg/m^3^) frequency shows a decreasing trend (1.3%: *ante-operam*; 0.1%: *in-operam*; 0.0%: *post-operam*, Table 3). This clear pollution reduction phenomenon can be generally due to the increasing use of catalyzed vehicles and the extension of limited traffic areas.

The effects of the infrastructural works during the *in-operam* period are quite evident, looking at the PM_10_ frequency distribution (Fig 3C). The whole curve relative to the *in-operam* period is skewed toward higher values compared to the curves of the *ante*- and *post-operam* periods. The maximum frequency in the *ante*- and *post*-*operam* periods is reached in the class of 44–66 μg/m^3^ (23.3% and 25.1%, respectively), while 23.4% of the values during the *in-operam* period are included in the class of 66–88 μg/m^3^. The frequency of lower concentration classes (<40 μg/m^3^) shows a minimum in the *in-operam* period (4.4% versus 11.7% in the *ante-operam* and 13.9% in the *post-operam*, Table 3). The *in-operam* period shows a higher average atmospheric concentration (97.5 μg/m^3^, vs 90.1 μg/m^3^ in the *ante-operam* and 84.3 μg/m^3^ in the *post-operam*, Table 2), and this is stressed also considering medium-high concentration classes (70–160 μg/m^3^) where frequency is 47.7% in the *ante-operam*, 59.2% in the *in-operam* and 45.1% in the *post-operam* (Table 3). The highest concentration classes (>200 μg/m^3^) are characterized by a decreasing trend from the *ante-operam* (4.3%) to the *in-operam* (3.7%) and to the *post-operam* (3.2%) (Table 3).

We applied the same procedure followed for Turin to our analysis of the city of Milan. For Milan we chose the air quality monitoring station of via Verziere. Like Turin’s Consolata station, Milan-Verziere is an Urban-Traffic station. This allowed us to make a comparison between the atmospheric concentration trends in the two areas and to evaluate how infrastructural work in Turin influenced air quality trends in an urban area generally influenced by road traffic emission levels.

As in Turin, at the Milan-Verziere station we obtained, for the period 2000–2008, the hourly atmospheric concentrations for NO_2_ and CO and the daily concentrations for PM_10_. We validated and divided the elementary data in the same concentration classes selected for Turin and we obtained the relative frequency histograms. In Table 4, we present the frequencies of concentration classes of elementary data and relative statistical information.

**Table 4:** Concentration class ranges and frequencies of elementary data for the three selected periods for Milan (*ante-operam*: 2000-2001-2002; *in-operam*: 2003-2004-2005; *post-operam*: 2006-2007-2008) and statistical information of concentration data.

NO_2_ –Hourly Air Concentration–MILAN				CO–Hourly Air Concentration—MILAN				PM_10_ –Daily Air Concentration—MILAN			
Conc. Classes - (μg/m^3^)	*Ante-Operam* Frequency Nr. Data	*In-Operam* Frequency Nr. data	*Post-Operam* Frequency Nr. data	Conc. Classes - (mg/m^3^)	*Ante-Operam* Frequency Nr. Data	*In-Operam* Frequency Nr. Data	*Post-Operam* Frequency Nr. Data	Conc. Classes - (μg/m^3^)	*Ante-Operam* Frequency Nr. Data	*In-Operam* Frequency Nr. Data	*Post-Operam* Frequency Nr. Data
**0–22**	334	27	429	**0.0–0.6**	4136	574	5689	**0–22**	139	21	232
**22–44**	3959	820	5241	**0.6–1.2**	8672	5135	12054	**22–44**	433	220	552
**44–66**	7754	4119	8516	**1.2–1.8**	5188	6026	5025	**44–66**	223	246	186
**66–88**	7599	8905	6659	**1.8–2.4**	4138	6604	1620	**66–88**	142	233	23
**88–110**	4060	7049	2884	**2.4–3.0**	1805	3336	234	**88–110**	77	157	4
**110–132**	1537	3072	711	**3.0–3.6**	995	2019	38	**110–132**	37	76	0
**132–154**	449	930	128	**3.6–4.2**	509	1052	15	**132–154**	21	55	0
**154–176**	173	326	15	**4.2–4.8**	281	546	0	**154–176**	11	34	0
**176–198**	77	149	0	**4.8–5.4**	131	285	8	**176–198**	2	8	0
**198–220**	25	47	0	**5.4–6.0**	100	225	0	**198–220**	4	8	0
**220–242**	16	24	0	**6.0–6.6**	51	124	0	**220–242**	4	17	0
**242–264**	4	0	0	**6.6–7.2**	24	52	4	**242–264**	1	4	0
**264–286**	5	4	0	**7.2–7.8**	20	48	0	**264–286**	0	0	0
**286–308**	1	4	0	**7.8–8.4**	11	32	0	**286–308**	1	4	0
**308–330**	2	8	0	**8.4–9.0**	8	20	0	**308–330**	0	0	0
**Nr. Data**	25995	25483	24583	**Nr. Data**	26069	26080	24686	**Nr. Data**	1096	1085	997
**Valid Data (%)**	99%	97%	93%	**Valid Data (%)**	99%	99%	94%	**Valid Data (%)**	100%	99%	91%
**Max**	328.3	305.8	346.8	**Max**	17.5	8.8	13.5	**Max**	293.0	186.0	185.0
**Mean**	71.3	58.6	54.6	**Mean**	1.5	1.2	1.3	**Mean**	54.1	50.9	47.7
**Std. Dev.**	28.8	26.4	29.8	**Std. Dev.**	1.0	0.8	0.7	**Std. Dev.**	36.7	27.3	30.7
**25**^**th**^ **percent.**	51.3	39.6	32.2	**25**^**th**^ **percent.**	0.8	0.6	0.8	**25**^**th**^ **percent.**	29.3	31.0	26.0
**Median**	68.4	56.2	49.9	**Median**	1.3	1.0	1.1	**Median**	43.0	44.0	38.0
**75**^**th**^ **percent.**	87.5	73.1	71.1	**75**^**th**^ **percent.**	1.9	1.6	1.7	**75**^**th**^ **percent.**	71.0	65.0	62.0
**95**^**th**^ **percent.**	121.6	105.6	107.3	**95**^**th**^ **percent.**	3.5	2.6	2.6	**95**^**th**^ **percent.**	127.0	105.0	113.0

We followed the same methodological approach that we used in Turin and we calculated the lognormal frequencies distribution of the concentration classes for the three selected pollutants for each of the three considered periods (*ante-operam*, *in-operam*, and *post-operam*). In Table 5 the statistical parameters of calculated lognormal distributions are shown.

**Table 5:** Parameters of calculated lognormal distributions related to the Milan data: *f(x)* ≅ *log* *N* (*μ*_*y*_ *; σ*_*y*_*)*.

	*Ante-Operam* 2000-2001-2002			*In-Operam* 2003-2004-2005			*Post-Operam* 2006-2007-2008		
	Lognormal distribution	*μ*_*x*_	*σ*_*x*_	Lognormal Distribution	*μ*_*x*_	*σ*_*x*_	Lognormal Distribution	*μ*_*x*_	*σ*_*x*_
**NO**_**2**_	f(x) = logN (4.18; 0.42)	71.70	31.64	f(x) = logN (3.96; 0.48)	59.16	30.35	f(x) = logN (3.85; 0.57)	55.31	34.32
**CO**	f(x) = logN (0.22; 0.64)	1.54	1.10	f(x) = logN (-0.07; 0.71)	1.19	0.96	f(x) = logN (0.12; 0.55)	1.31	0.77
**PM**_**10**_	f(x) = logN (3.80; 0.61)	53.95	36.57	f(x) = logN (3.79; 0.53)	51.17	29.20	f(x) = logN (3.67; 0.64)	48.14	34.01

**Fig 4:**
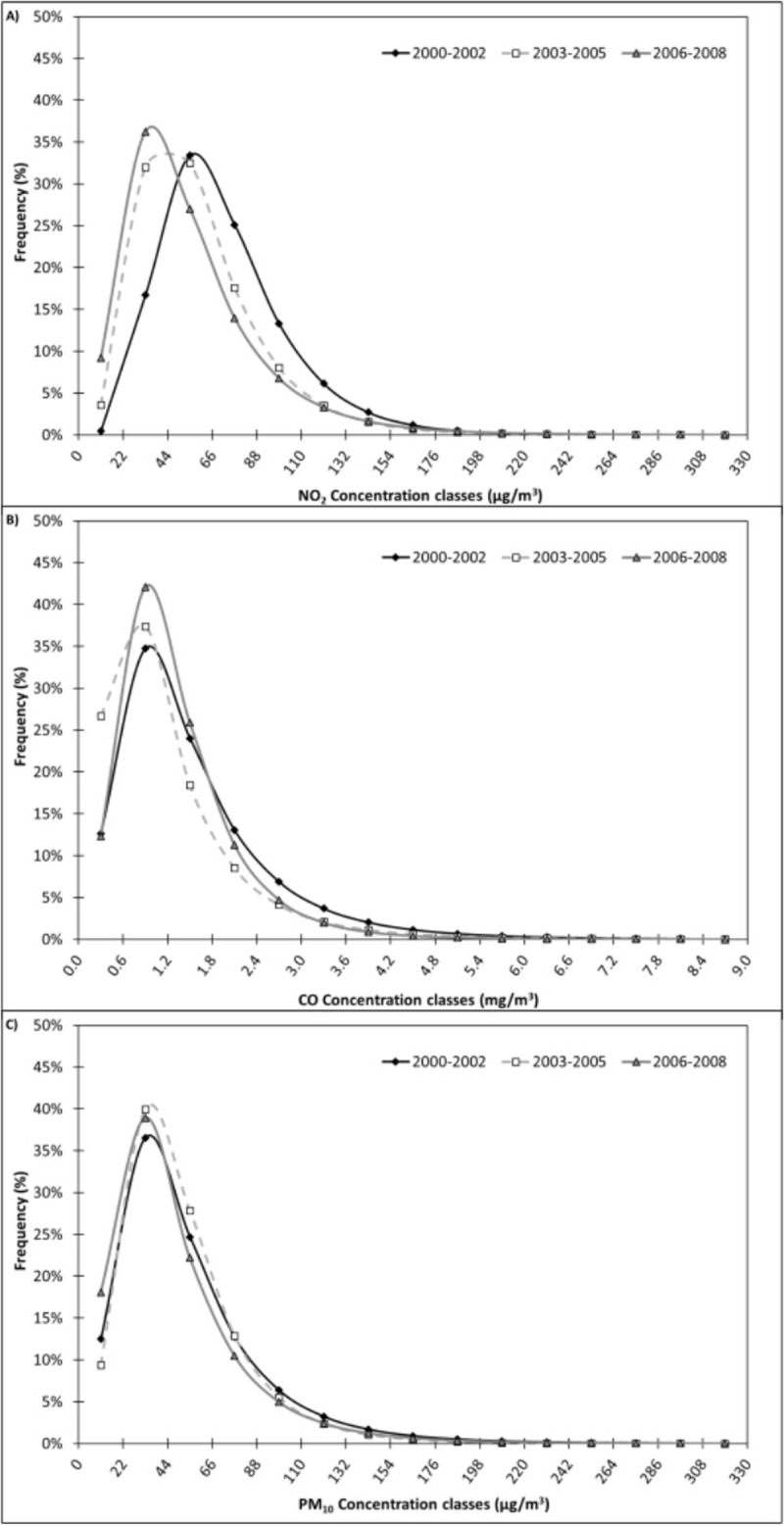
Re-calculated frequency distribution of NO_2_ (A), CO (B) and PM_10_ (C) sorted according to 15 concentration classes recorded in Milan in each of the three periods (*ante-operam*, *in-operam*, and *post-operam*).

From calculated Lognormal distributions, it was possible to obtain the re-calculated frequency of each atmospheric concentration class and to plot the frequency of the 15 selected classes in each of the three periods (*ante-operam*, *in-operam*, and *post-operam*), obtaining the graphs of frequency distributions, as shown in Fig 4.

Firstly, a different trend between the Turin and Milan NO_2_ datasets can be observed (Figs 3A and 4A, respectively). In Turin, the highest value of average concentrations is registered in the *in-operam* period, while in Milan, a decreasing trend is clearly defined along the whole period (71.7 μg/m^3^: *ante-operam*; 59.2 μ g/m^3^: *in-operam*; 55.3 μg/m^3^: *post-operam*, Table 5). Considering the frequency of concentration classes, in Milan the curves are clearly skewed towards lower concentration classes from the *ante-operam* to *post-operam* periods (Fig 4A), while in Turin the curves feature similar shapes (Fig 3A). The highest frequency is registered for the 44–88 μg/m^3^ concentration class during the *ante-operam* period (58.5%) and for the 22–66 μg/m^3^ concentration class during the *in*- and *post-operam* periods (64.5% and 63.2%, respectively). In Milan, lower concentration classes (<40 μg/m^3^) clearly show an increasing trend in frequency along the whole period (12.0%: *ante-operam*; 28.5%: *in-operam*; 38.9%: *post-operam*, Table 6), while in Turin, these concentration classes show the lowest frequency during the *in-operam* period (16.7% vs 19.4% in the *ante-operam* and 21.5% in the *post-operam*, Table 4). In Milan, medium-high concentration classes (70–160 μg/m^3^) are characterized by frequencies with a clear decreasing trend along the whole period (42.2%: *ante-operam*; 26.7%: *in-operam*; 22.6%: *post-operam*, Table 6), while in Turin, the maximum was found during the *in-operam* period (39.5% vs 35.6% in the *ante-operam* and 36.3% in the *post-operam*, Table 4). In Milan, the classes with the highest concentrations (>160 μg/m^3^) feature a vague increasing trend along the whole period, unlike the results obtained from the Turin dataset: 1.7%, 1.1% and 1.6% from the *ante*- to the *in*- and *post*-*operam* periods (Table 6).

**Table 6:** Re-calculated frequency distribution of NO_2_, CO and PM_10_ sorted according to 5 concentration classes recorded in Milan in each of the three periods (ante-operam, in-operam, and post-operam).

NO_2_ –Hourly Air Concentration– Lognormal Distribution—MILAN				CO–Hourly Air Concentration Lognormal Distribution—MILAN				PM_10_ –Daily Air Concentration Lognormal Distribution—MILAN			
Conc. Classes (μg/m^3^)	*Ante-Operam* Frequency	*In-Operam* Frequency	*Post-Operam* Frequency	Conc. Classes (mg/m^3^)	*Ante-Operam* Frequency	*In-Operam* Frequency	*Post-Operam* Frequency	Conc. Classes (μg/m^3^)	*Ante-Operam* Frequency	*In-Operam* Frequency	*Post-Operam* Frequency
**0–40**	12.0%	28.5%	38.9%	**0–1**	36.3%	54.0%	41.2%	**0–40**	42.9%	42.2%	51.1%
**40–70**	44.1%	43.7%	36.9%	**1–2**	40.4%	32.0%	44.1%	**40–70**	33.9%	38.1%	30.7%
**70–110**	32.9%	21.4%	17.4%	**2–5**	21.7%	13.1%	14.4%	**70–110**	16.1%	15.2%	12.9%
**110–160**	9.3%	5.3%	5.2%	**5–7**	1.2%	0.6%	0.3%	**110–160**	5.2%	3.6%	3.9%
**160–200**	1.3%	0.8%	1.0%	**7–10**	0.3%	0.2%	0.0%	**160–200**	1.2%	0.6%	0.8%
**>200**	0.4%	0.3%	0.6%	**>10**	0.1%	0.0%	0.0%	**>200**	0.7%	0.2%	0.5%

Secondly, as for CO concentrations, we can appreciate similar trends between Turin (Fig 3B) and Milan (Fig 4B). As regards average concentrations, in both cities the highest value is registered in the *ante-operam* period, but the clear decreasing trend along the whole period registered in Turin is not present in Milan (1.5 mg/m^3^: *ante-operam*; 1.2 mg/m^3^: *in-operam*; 1.3 mg/m^3^: *post-operam*, Table 5). The concentration classes in Milan feature similar frequency curves to those also found in Turin (Fig 4B). In all three periods, the most frequent concentration class is 0.6–1.2 mg/m^3^ with a clear increasing trend throughout; however, in Milan differences among the frequencies of the three periods are less evident. Similarly to Turin, lower concentration classes (< 1mg/m^3^) do not show a clear increasing trend and the highest frequency is found to occur in the *in-operam* period (Table 6). The clear decreasing trend for medium-high concentration classes (2–7 mg/m^3^) registered in Turin (Table 4) is not found with the Milan dataset (Table 6). In Turin, the frequency of these concentration classes ranged from 36.8% (*ante-operam*) to 21.4% (*in-operam*) to 8.9% (*post-operam*), while in Milan the values are 22.9%, 13.7% and 14.7%, respectively. As with Turin, high concentration classes (>7 mg/m^3^) clearly show a decreasing trend.

Finally, for PM_10_ concentrations we can find different trends between Turin (Fig 3C) and Milan (Fig 4C), as with the NO_2_ dataset (Figs 3A and 4A). In general, higher concentrations were registered in Turin (average concentrations range from 84.3 to 97.5 μ g/m^3^ in Turin, Table 2, and from 48.1 to 54.0 μg/m^3^ in Milan, Table 5). While in Turin, the highest value of average concentrations is registered in the *in-operam* period, in Milan we observed a clear decreasing trend along the whole period (54.0 μg/m^3^: *ante-operam*; 51.2 μ g/m^3^: *in-operam*; 48.1 μg/m^3^: *post-operam*, Table 5). Regarding concentration classes frequencies, the *in-operam* curve of the Turin dataset is skewed towards higher concentration classes (Fig 3C), while in Milan the curves of the three periods are similar (Fig 4C). In Milan, the peaks in all three periods are in the 22–44 μg/m^3^ class (36.6%, 40.0% and 38.9% for the *ante*-, *in*- and *post-operam* periods, respectively), all concentrations lower than in the Turin dataset. The 2006–2008 years are characterized by higher frequencies of lower concentrations. This suggests a decreasing trend of pollution levels not observed in Turin, probably due to the impact of the infrastructural work for the Olympic Games. Lower concentration classes (< 40 μg/m^3^) do not show a clear trend in frequency. As with Turin, the *in-operam* period shows the lowest frequency even if the differences are less evident in the Milan dataset. In particular, in Turin the frequency is 4.4% (vs 11.7% and 13.9% for the *ante-operam* and *post-operam* periods, Table 3), while in Milan, the frequency is 42.2% (vs 42.9% and 51.1% for *ante-operam* and *post-operam* periods, Table 6). Medium-high concentration classes (70–160 μg/m^3^) show a clear decreasing trend in Milan along the whole period (21.3%: *ante-operam*; 18.8%: *in-operam*; 16.8%: *post-operam*, Table 6) that was not registered in Turin. In Turin, the frequency of these classes ranged from 47.7% (*ante-operam*) to 59.2% (*in-operam*) to 45.1% (*post-operam*), showing the maximum in the *in-operam* period (Table 3).

**Fig 5:**
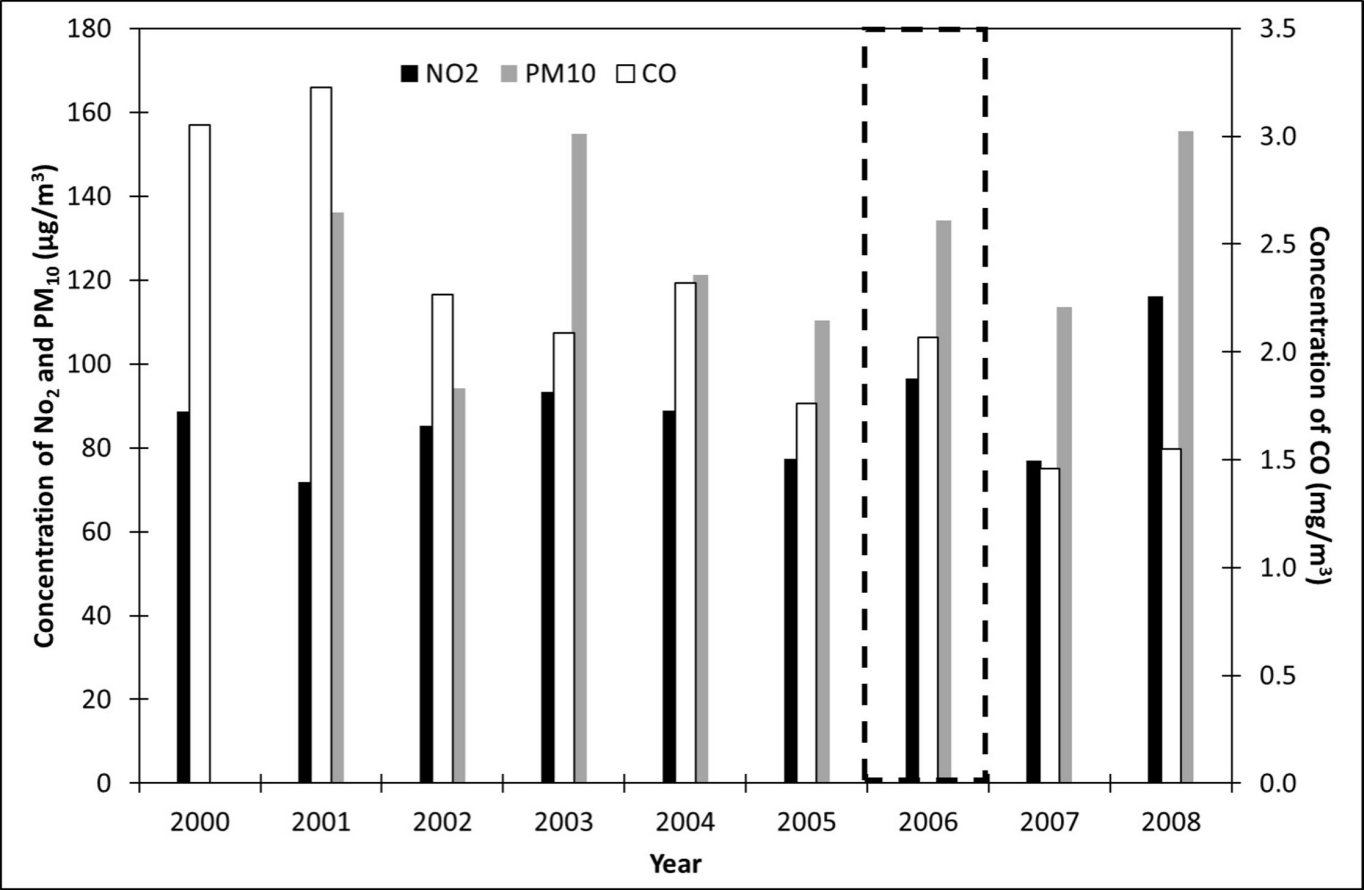
Concentration values of NO_2_, CO and PM_10_ averaged from 10 to 26 February of each year (from 2000 to 2008). The data for the year 2000 are missing. The days with Turin Winter Olympics are marked with a dotted black box.

In addition to the 3-year period when all infrastructures needed for the organization were built (2003–2005), we also focused on the 2-week period of the Turin Winter Olympics (from 10 to 26 February 2006), comparing it with the same days from 2000 to 2008 (Fig 5). As the period under examination is too short to perform a statistical analysis of the concentration class frequencies, we considered the averages and the 95^th^ percentile values. The average concentration of atmospheric NO_2_ in 2006 (96 μg/m^3^) shows the second highest value, after 2008 (116 μg/m^3^), and it is +8 μg/m^3^ above the average value registered in the same period throughout the 9 years (88 μg/m^3^). Even considering the 95^th^ percentile, this year turns out to show the second highest values, after 2008 (159 and 192 μg/m^3^in 2006 and 2008, respectively), and it is +24 μg/m^3^ above the average value registered in the same period throughout the 9 years (134 μg/m^3^). As far as CO is concerned, the atmospheric concentration is lower compared to the previous years (i.e. *ante*- and *in-operam*), but higher than the values of the following 2 years (i.e. *post-operam*). Therefore, even if the infrastructural projects were finished and the mitigation policies in limiting traffic had been adopted since 2004, the CO atmospheric concentration in 2006 showed the highest value of the *post-operam* period. Therefore, it could be true that the activities for the Olympic Games may have determined higher atmospheric concentration levels of NO_2_ and CO. However, it is important to consider that air pollution at any point in time is the result of many diverse meteorological, physiographic and emission factors acting simultaneously. Consequently, weather conditions could have affected the registered values: with the same emissions, for example wind and rain can lead to different levels of concentration. This issue does not concern the *in-operam* analyses because the long series of data (3 years) allows us to implicitly consider the meteorological conditions. The PM_10_ pattern is not as evident as those of NO_2_ and CO, so it is not possible to determine clearly if 2006 was influenced by the Olympic Games.

## Discussion and conclusions

We analyzed the atmospheric concentration data for NO_2_ and CO (hourly samples) and PM_10_ (daily samples) over a nine-year period (2000–2008) in the two most important cities of Northern Italy, Turin and Milan. We chose two atmospheric air quality monitoring stations of the same type (i.e. Urban-Traffic station): Turin-Consolata and Milan-Verziere. In this way, we could define how the infrastructural work for the Turin 2006 Olympic Games modified the air pollution trends over time (Turin), compared to a similar urban area not affected by any type of infrastructural work (Milan). Since the infrastructural construction covered a three-year period (2003-2004-2005: *in-operam*), we decided to compare this period to a time frame of three years before (2000-2001-2002, corresponding to the *ante-operam*) and three years after (2006-2007-2008, corresponding to the *post-operam*).

To evaluate the reliability of the lognormal distribution chosen for describing the environmental concentrations of atmospheric pollutants, we verified the “goodness-of-fit” between observed and theoretical data, applying the Chi-Square Test. This test passed for all the data vectors we used in this study, verifying the well-known assumption that pollutant concentrations in environmental media follow a lognormal statistical distribution [24]. We found χ2 (i.e. the sum of the squared differences between the empirical and the theoretical frequencies of each class of values) to be higher than r (i.e. the critical values of Chi-Square table) for each of all the 9 data vectors we used in this study, confirming with a probability of 95% that atmospheric concentrations of NO_2_, CO and PM_10_ follow a lognormal statistical distribution. Finally, the lognormal distribution turns out to be the best fitting statistical distribution for all datasets.

We found out that infrastructural work in Turin affected the NO_2_ atmospheric concentrations, determining a peak in the average values during the *in-operam* period. This pattern did not emerge from Milan data where a decreasing trend was evident from the *ante-operam* to the *post-operam* period. This observed decreasing trend is probably due to: i) the progressive rise in the use of less polluting vehicles (the emission factors are 2100 mg/km for Gasoline Pre EURO, 80 mg/km for Gasoline EURO 3 from 2001, 48 mg/km for Gasoline EURO 4 from 2005, and 914 mg/km for Diesel Pre EURO, 803 mg/km for Diesel EURO 3 from 2001, 602 mg/km for Diesel EURO 4 from 2005, (data from ARPA Lombardia); ii) the continuous transformation of civil heating systems by oil, diesel, natural gas (the emission factors are 860 mg/kWh, 200 mg/kWh, and 90 mg/kWh, respectively, from ARPA Lombardia); and iii) the transformation of thermoelectric power stations from the single cycle steam to combined cycle gas (the emission factors are 1400 mg/kWh at 260/160 per combined cycle, from ARPA Lombardia). We can conclude that the Olympic infrastructural projects modified this decreasing trend in Turin determining a peak in average atmospheric concentrations during the working period (2003–2005). For the same reason, the shift of the frequency curves towards lower concentration classes that we observed in Milan (the most frequent class changed from 66–88 μg/m^3^ in the *ante-operam* period to 22–44 μg/m^3^ in the *post-operam* period), is not present in Turin, where the curves do not show well defined differences among the three periods (in all three periods, the most frequent concentration class is 44–66 g/m^3^).

CO concentration classes show a similar frequency trend between Turin and Milan. The most frequent concentration class in both the cities is 0.6–1.2 mg/m^3^ but Turin shows a more distinct decreasing trend related to average values and to frequencies of medium-high concentration classes (2–7 mg/m^3^).

The generalized reduction in CO levels found in both cities can be linked to the increasing use of catalyzed vehicles (the emission factors are 9 g/km (Pre EURO), 1.5 g/km (EURO 3, from 2001), and <0.6 g/km (Diesel), from ARPA Lombardia). The decreasing trend more evident in Turin compared to Milan can be due to the simultaneous expansion of limited traffic areas created to carry out the infrastructural work.

The effects of the infrastructural work during the *in-operam* period are quite evident even in the PM_10_ concentration frequency distribution. While in Turin, the highest value in average concentration is registered in the *in-operam* period, in Milan we observed a clear decreasing trend throughout the whole period. This general decrease in PM_10_ atmospheric concentrations may have been determined by new environmental policies that forced industries to adopt the best available technologies (IPPC CE Directives), reducing emissions of primary pollutants (i.e. sulfur oxides and nitrogen oxides), as well as the renewal of vehicles (from ARPA Lombardia). In Milan, the most frequent concentration class is 22–44 μg/m^3^ in all the three periods, unlike in Turin, where the most frequent concentration class was found to be skewed from 66–88 μg/m^3^ (*ante-operam* period) to 44–66 μg/m^3^ (*in-operam* period). Medium-high concentration classes (70–160 μg/m^3^) showed a clear decreasing frequency trend in Milan throughout the whole period (21.3%: *ante-operam*; 18.8%: *in-operam*; 16.8%: *post-operam*) that was not registered in Turin, where the peak frequency of medium-high concentration classes is registered in the *in-operam* period (59.2%). In the same period, in Turin a peak in the average value of concentrations (97.5 μg/m^3^) was registered. The general decreasing trend in PM_10_ concentrations registered in Milan was not observed in the Turin dataset, probably due to the high impact of infrastructural work that determined a higher emission of fine dust from different sources and with different profiles (road traffic for Milan and caterpillar emissions and excavation work for Turin).

From this preliminary analysis, we can conclude that the infrastructural work for the Olympic Games influenced the air quality levels related to NO_2_ and PM_10_ concentrations in Turin. No negative effects were observed regarding the CO dataset. This evidence can be linked to the adoption of effective urban environmental policies from 2000 to 2008 (i.e. less polluting vehicles, higher efficiency civil heating systems, and more road traffic limitations). The negative effects on NO_2_ and PM_10_ levels can be linked to the consideration that during the period of infrastructural work, more constant and higher emission levels occurred in Turin (due to caterpillar tractor emissions) with an emission profile that is different from the classic road traffic emission profile. This generally leads to a reduction of extreme and isolated concentration values and to higher average atmospheric concentrations. Within this contest, the councils of cities that are going to hold the Olympic Games should adopt measures to reduce air pollution during the infrastructural work for the event, i.e. avoiding road traffic in the inner areas or limiting traffic areas. In this way, as the experience of Turin 2006 shows, at least CO levels would be limited. Moreover, traffic represents one of the most relevant sources of emissions even for other pollutants, therefore more attention should be given to this issue, not only during the days of the Olympic event but also during the previous years when all infrastructures needed for the organization would be built.
